# Understanding rapid implementation from discovery to scale: Rwanda’s implementation of rotavirus vaccines and PMTCT in the quest to reduce under-5 mortality

**DOI:** 10.1186/s12887-023-03888-4

**Published:** 2024-02-28

**Authors:** Felix Sayinzoga, Lisa R. Hirschhorn, Jovial Thomas Ntawukuriryayo, Caroline Beyer, Kateri B. Donahoe, Agnes Binagwaho

**Affiliations:** 1 Rwanda Biomedical Center, Kigali, Rwanda; 2 University of Global Health Equity, Kigali, Rwanda; 3 Northwestern University Feinberg School of Medicine, Chicago, USA

**Keywords:** Under-5 mortality, Maternal and child health, Implementation research, Rwanda

## Abstract

**Background:**

Over the last eight decades, many evidence-based interventions (EBIs) have been developed to reduce amenable under-5 mortality (U5M). Implementation research can help reduce the lag between discovery and delivery, including as new EBIs emerge, or as existing ones are adapted based on new research. Rwanda was the first low-income African country to implement the rotavirus vaccine (RTV) and also adopted Option B+ for effective prevention of mother-to-child transmission (PMTCT) before the World Health Organization’s (WHO) recommendation. We use implementation research to identify contextual factors and strategies associated with Rwanda’s rapid uptake of these two EBIs developed or adapted during the study period.

**Methods:**

We conducted a mixed methods case study informed by a hybrid implementation research framework to understand how Rwanda outperformed regional and economic peers in reducing U5M, focusing on the implementation of health system-delivered EBIs. The research included review of existing literature and data, and key informant interviews to identify implementation strategies and contextual factors that influenced implementation outcomes. We extracted relevant results from the broader case study and used convergent methods to understand successes and challenges of implementation of RTV, a newly introduced EBI, and PMTCT, an adapted EBI reflecting new research.

**Results:**

We found several cross-cutting strategies that supported the rapid uptake and implementation of PMTCT, RTV, and leveraging facilitating contextual factors and identifying and addressing challenging ones. Key implementation strategies included community and stakeholder involvement and education, leveraging of in-country research capacity to drive adoption and adaptation, coordination of donors and implementing partners, data audit and feedback of coverage, a focus on equity, and integration into pre-existing systems, including community health workers and primary care. The availability of donor funding, culture of evidence-based decision-making, preexisting accountability systems, and rapid adoption of innovation were facilitating contextual factors.

**Conclusion:**

Implementation strategies which are generalizable to other settings were key to success in rapidly achieving high acceptability and coverage of both a new and an evolving EBI. Choosing strategies which leverage their facilitating factors and address barriers are important for other countries working to accelerate uptake of new EBIs and implement needed adaptations based on emerging evidence.

## Introduction

The United Nations Millennium Development Goal 3 called for the reduction of under-5 mortality (U5M) by two-thirds between 2000 and 2015. As countries prioritized this reduction, significant progress was observed in many countries. This progress was supported by the development of evidence-based interventions (EBIs) proven to reduce deaths in children under 5 [1]. However, the implementation of these EBIs at scale and with quality and equity has lagged behind, hindering countries’ goals to reduce preventable U5M through a quality and effective health system [2–4].

Implementation research is defined as “the scientific study of the use of strategies to adopt and integrate evidence-based health interventions into clinical and community settings to improve individual outcomes and benefit population health” [5]. This area of study bridges the knowledge gap between effectiveness of EBIs and the how, why, when, and where of their uptake into health systems. Implementation research is important in understanding successes and challenges in implementing new EBIs in low- and middle-income countries (LMICs) [6–9]. However, much of the literature on EBIs focuses on effectiveness and success in achieving coverage (sometimes with quality). These papers do not often capture important insights into what was done (implementation strategies) and what worked and why, including barriers and facilitators which may have influenced strategy choice and success (contextual factors). As new EBIs and evidence on adapting implemented EBIs emerge, understanding how countries are successful in accelerating the uptake and integration of new innovations into health systems is key to closing the distance between research and practice.

To help bridge this gap, between 2017 and 2020, we conducted implementation research case studies on six countries (Rwanda, Nepal, Senegal, Peru, Bangladesh, and Ethiopia) that outperformed their geographic and economic peers in dropping U5M between 2000 and 2015. We developed a hybrid implementation research framework, building on existing frameworks to guide our study in identifying implementation strategies and contextual factors that served as barriers or facilitators in LMICs relevant to amenable U5M; and exploring implementation outcomes such as coverage, feasibility, acceptability, and fidelity. This framework shows the interconnectedness in how EBIs are implemented from exploration to preparation, implementation, adaptation, and sustainment (EPIAS), with use of implementation strategies adapted to contextual factors to influence the implementation outcomes [10].

Since the 1994 Genocide against the Tutsi that took approximately one million lives, destroyed existing public health infrastructure and human resources, and left the country one of the poorest in the world, Rwanda has made significant strides in improving key markers of improved human development, including U5M. The country prioritized good governance with well-structured decentralization of leadership from national to community levels, and improved health systems, including coverage of qualified healthcare workers and development of a strong community health worker (CHW) program to strengthen the quality of healthcare services [11, 12]. U5M decreased from 196 deaths per 1000 live births in 2000 to 50 deaths per 1000 live births in 2015 [13]. Similarly, neonatal mortality decreased, although at a slower rate, from 44 deaths per 1000 live births to 20 deaths per 1000 live births in the same period [13]. A significant contributor to this success was the rapid uptake, expansion, and adaptation of existing and emerging EBIs.

In this paper, we use the case study from Rwanda to explore how the country was able to accelerate the implementation of one new EBI, rotavirus vaccines (RTV), and one existing EBI where new evidence informed adaptation, prevention of mother-to-child transmission of HIV (PMTCT). We focus on understanding the steps Rwanda took to explore, prepare, implement, adapt, and sustain the EBIs, the strategies adopted and adapted at each step of the process, and contextual factors that influenced the choice of these implementation strategies and either facilitated or hindered the progress towards equitable and quality implementation [14]. The paper provides a better understanding of how Rwanda was able to identify, integrate, and deliver new or evolving EBIs rapidly and equitably, providing important lessons for other countries looking to accelerate implementation of new EBIs and adapt existing ones as new evidence for improving effectiveness emerges through research and experience.

## Methods

### EBIs

We identified EBIs known to reduce the most common causes of U5M among infants and children in LMICs, which existed or were introduced during the study period, through a review of existing literature and guidelines from Millennium Development Goal (MDG) efforts. This list of EBIs guided the evidence review and selection of key informants, as well as the exploration of implementation strategies, contextual factors, and implementation outcomes [10, 15].

To explore the timeliness from research findings on effectiveness and availability of implementation in Rwanda, we chose two EBIs that either had emerging evidence requiring adaptation or that evolved during the study period. Further, these were chosen to represent different implementation barriers and facilitators. Reducing morbidity and mortality due to the rotavirus required a strong vaccine supply chain and three visits integrated into an existing vaccination schedule, while PMTCT required integration into existing maternal and HIV systems of care, addressing stigma, and ensuring continuity before, during, and after pregnancy.

WHO began recommending rotavirus vaccines to be used in all national child immunization programs in 2009 [16] and consequently the Rwandan Ministry of Health (MOH) after studies done by the University Teaching Hospital of Kigali (CHUK) in 2010–2011 showed high burden of severe diarrhea caused by rotavirus. Implementation of this vaccine started in 2012 with the support of GAVI funding [17].

Effective PMTCT (defined as combined antiretroviral therapy (ART) for HIV-positive women before, during, and after pregnancy) was piloted by the MOH Kigali health facilities in 1999 [18], before it was recommended by WHO in 2000 [19]. This was done in accordance with international data and national protocol, after collecting data over the course of 10 years on the burden of HIV at CHUK, which had been testing for HIV among pregnant women since 1989. The program was scaled up in 2006.

### Data collection

We extracted relevant information regarding the implementation of RTV and PMTCT from the full case study on Rwanda. The methods used for the case study are summarized below.

#### Desk review of documents and literature

For the case study, we conducted a search of peer-reviewed literature on rates and progress towards reduction of amenable U5M in Rwanda, focusing on uptake and implementation of EBIs. The search was done through MEDLINE (PubMed) and Google Scholar using key search terms such as “child mortality” or “under-five mortality” and “Rwanda,” specific EBIs (e.g., “rotavirus vaccine”), causes of U5M (e.g. “diarrhea”), and contextual factors (e.g. “conflict”). We examined other sources, including national policies and reports, Countdown to 2015, and other available reviews of U5M reduction work in Rwanda, including reports on WHO maternal and child health initiatives [15, 20–22]. The review looked for reports and articles that described strategies, contextual factors, policies, and coverage of the U5M-targeted health system-delivered EBIs between 2000 and 2015. We also reviewed bibliographies of identified articles and progressively updated the search as additional relevant sources were identified.

#### Quantitative data

We used the Demographic and Health Surveys (DHS) to extract quantitative data on EBI coverage, reported childhood illnesses, and measures of equity.

#### Key informant interviews

For the case study, we interviewed a purposive sample of 16 key informants (KIs) from organizations that had been involved with implementation of the EBIs between 2000 and 2015, including former or current employees of the Rwanda MOH, non-governmental organizations, donor organizations, and other partners. The KI selection was not designed for saturation but instead was designed to comprehensively cover EBIs, including RTV and PMTCT, and was limited by time and resources. Using a semi-structured interview guide based on the implementation research framework, the interviews were conducted in a manner that addressed the complete EBI implementation process, from exploration to sustainment. Follow-up interviews were conducted as needed when gaps in understanding of implementation strategies, outcomes and contextual factors were identified from desk review or other KI interviews. Key informants helped identify additional data sources to deepen our understanding of implementation and outcomes. All interviews except one were led by one of the project leads (LRH), with one to two notetakers.

#### Analysis

For the case study, we used an explanatory mixed methods approach with directed content analysis of KI interviews based on the framework [23], to identify implementation strategies and contextual factors for the EBIs, as well as facilitators and barriers at the local, national, and global levels. We used the framework to develop an initial set of codes for EBIs, implementation outcomes, and contextual factors. Coding was done manually with additional codes added based on identification of new concepts, factors, or strategies. Guided by the framework, we also extracted evidence of the implementation outcomes for EBIs from the available literature and quantitative data, including appropriateness, acceptability, feasibility, effectiveness, coverage or reach, and equity, where possible. These results were analyzed by the team and reviewed through a stakeholder meeting in Rwanda to provide feedback and validation, and identify transferable lessons with the potential for adoption or adaptation in other countries with challenges in reducing U5M.

### Ethical considerations

This research was approved by and conducted with the support of the Institutional Review Board of the University of Global Health Equity, the Ministry of Health, National Health Research Committee, and Rwanda Biomedical Center’s Division of Maternal, Child and Community Health. Informed consent was sought and obtained from all interview participants.

## Results

When rollout of rotavirus vaccine began in May 2012, Rwanda became the first low-income African country to introduce the vaccine. The coverage of three doses of the vaccine rapidly increased from 50% in 2012 to 99% in 2013 [24], decreasing slightly to 95% in 2015 [25]. Equity in subnational coverage was high, ranging from 94% in Southern and Western Provinces to 96% in Eastern Province and Kigali City. Similar results were seen based on wealth: 91% in the lowest quintile versus 96% in the highest (wealthiest) quintile [25]. PMTCT coverage also expanded, with a steady increase in health facilities offering PMTCT, from 53 health facilities in 2003 to 382 in 2010. By 2012, 97% (488) of all health facilities (hospitals and health centers) offered PMTCT [26]. In terms of uptake, between 2004 and 2015, the percentage of pregnant women with HIV who received antiretroviral medicine for PMTCT increased from 33 to 100% [27]. Importantly, PMTCT intervention was adapted to reflect updated research evidence and maximize the benefit to the community.

### Key strategies that facilitated implementation and maintenance of rotavirus vaccination

**Fig. 1:**
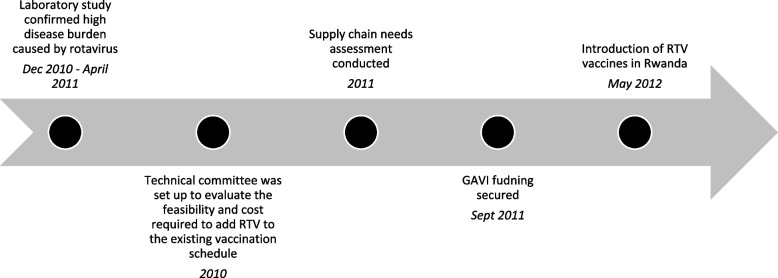
Timeline of the implementation of rotavirus vaccine (RTV) in Rwanda

Rotavirus vaccination was introduced to reduce diarrhea-related deaths among children under 5. With the use of a number of implementation strategies (Table 1), three doses of RTV were routinely administered at 6, 10, and 14 weeks after birth, as recommended by WHO [28]. Key steps in the implementation process are summarized in Fig. 1.

**Table 1:** Implementation strategies for rotavirus vaccine (RTV) and prevention of mother-to-child transmission (PMTCT)

Implementation Strategies	RTV	PMTCT
Rapid and early adoption of innovations	X	X
Stakeholder engagement (local)	X	X
Stakeholder engagement (international)	X	X
Rapid scale-up	–	X
Phased scale-up	–	–
Data use to understand disease burden	X	X
Small-scale testing	–	X
Data use for prioritization	X	X
Development and adaptation of protocols & guidelines	X	X
Community engagement	X	X
Training	X	X
Community education and sensitization	X	X
Leveraging donor support	X	X
Supportive supervision and mentoring	–	–
Surveillance use	X	X
Data systems strengthening	–	X
Cold and supply chain strengthening	X	–
Integration into systems (adding on)	X	X
Task shifting	–	X
Data use for adaptation	X	X
Public-private partnership (PPP)	–	–
Community-based care delivery	–	–
Multi-sector approach	–	–
Human resources for health expansion	–	–
Leveraging existing systems (to implement a strategy)	X	X
Government financing of evidence-based intervention	X	X
Focus on equity	X	X
National leadership and accountability	X	X
Donor and implementing partner coordination	X	X
Prioritization of neonatal mortality	–	–
Data use for decision-making	X	X
Focus on improving quality	–	–

#### Exploration (E)

Rwanda used nationally-produced research findings to understand the burden of diarrhea associated with rotavirus and decide if RTV would be an appropriate intervention for the country. Between December 2010 and April 2011, the laboratory at CHUK found that 30% of pediatric patients hospitalized for severe diarrhea tested positive for rotavirus, which was a high burden sufficiently compelling to argue for the need to adopt RTV [17].

#### Preparation (P)

The previous success of the pneumococcal conjugate vaccine (PCV) rollout and analysis of immunization training, supply chains, and surveillance informed the strategies for introduction of RTV in Rwanda. In 2010, Rwanda established a technical committee to evaluate the feasibility and cost required to add rotavirus to the existing vaccination schedule [17]. A supply chain needs assessment was conducted using the WHO Logistics Forecasting Tool to identify the necessary cold chain development at the district and facility level required to accommodate the new vaccine. The assessment found that 120 of the 426 primary health centers with immunization activities needed additional cold chain capacity. The government applied for support from GAVI in June 2011 and the funding was secured 3 months later [29]. A number of strategies were also utilized to ensure acceptability, effectiveness, and sustainability (Table 2).

**Table 2:** Rotavirus vaccine implementation strategies and outcomes

Outcomes	Implementation strategy	Results
Acceptability	Community education and engagement and advocacy – through community volunteers, with key messages; engagement of stakeholders.	High coverage rates reflecting community acceptance; per a KI, “*It was very easy to administer the vaccine because mothers were not afraid of it*.”
Feasibility	Gaps in cold chain management were identified and addressed; close follow-up with engaged partners; training of community health workers; clear vaccination schedule.	Rotavirus vaccine was delivered nationally.
Fidelity	Revise EPI data collection tools, integration into existing schedule [17].	Three doses of rotavirus vaccine given at 6, 10, and 14 weeks as recommended [28].
Effectiveness and Coverage	Health facility-specific timelines for roll-out were determined; cold and supply chain strengthening undertaken; task-shifting by initiating antiretroviral therapy by nurses at health centers.	Rotavirus vaccination in children under 1 increased from 50% in 2012 to 99% in 2013 [24]. Hospital admissions for diarrhea in children under 5 fell by 49% between 2011 and 2013 [24].
Equity	RTV was integrated into the EPI system, with delivery free of charge in all parts of the country and use of motorcycles to take vaccines to children in remote areas.	By 2015, subnational coverage ranged from 94% in the Western and Southern Provinces to 96% in the Kigali City and Eastern Province. The coverage was also 91% in the poorest and 96% in the richest [25].

*EPI* Expanded Program on Immunization, *RTV* rotavirus vaccine

#### Implementation (I)

Coordination with donor and implementing partners was a key strategy that facilitated the implementation of RTV. Leveraging donor funding while committing national resources was also critical. GAVI funded the rollout, with the remaining costs (counterpart funding) covered by the Government of Rwanda [17]. Additional strategies included integration of rotavirus vaccination into the routine immunization program and national rollout early in the process. Education and sensitization through community volunteers supported local understanding of rotavirus vaccine benefits and built on previous community experience of successful vaccines as an important contextual facilitator. One KI noted that it was very easy to administer the vaccine because mothers were not afraid of it as they had already seen the success of previous vaccinations and wanted to see similar success with diarrhea, especially given that the vaccine was being implemented during diarrheal season. The KI also noted that messaging was streamlined because there is one common language in Rwanda and that training of health workers contributed to the success of the rollout.

The country’s focus on equity resulted in the delivery of the rotavirus vaccine in all parts of the country. This was done through financial protection (delivery of vaccines free of charge), geographic access to healthcare for even the most remote areas (for example, through use of motorcycles to allow for health worker transport to children in remote areas near their homes), planning from the start for nationwide delivery, and data review to identify and focus on reaching those who were not benefiting from the program.

#### Adaptation (A)

To ensure ongoing data use for decision-making and accountability, the MOH coordinated the collection of data and the monitoring and evaluation efforts to track progress and gaps and to implement corrective actions in a timely manner. The CHWs and local health centers collected data and, via the district hospitals, sent them to the MOH, where they were analyzed to provide feedback and guidance for improvement to the reporting entities.

#### Sustainment (S)

As a result of these strategies, and facilitating contextual factors discussed below, this vaccine was successfully integrated into the existing routine vaccination system. The MOH continued the integrated monitoring of coverage, maintained the national supply chain, and secured the counterpart funding in the annual national budget. In 2015, 95% of children aged 12–23 months had received all three doses of the rotavirus vaccination [25].

### Key strategies that facilitated implementation and maintenance of prevention of mother-to-child transmission (PMTCT)

**Fig. 2:**
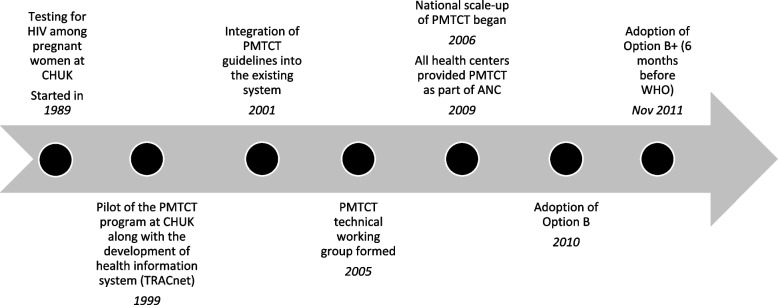
Timeline of the implementation of prevention of mother-to-child transmission (PMTCT) in Rwanda

Rwanda used several implementation strategies (Table 1) to implement and sustain delivery of PMTCT as part of HIV services for women and preventive services for children under 5. During antenatal care (ANC) services, pregnant women and their partners received HIV counseling, underwent testing if they agreed to do so, and women who tested positive were given antiretroviral (ARV) prophylaxis. During ANC, women were also routinely educated about HIV prevention and treatment to make sure the risk of HIV infection and transmission from mother to her baby was minimized [18]. Importantly, Rwanda was one of the first countries in sub-Saharan Africa to implement emerging research identifying more effective regimens for PMTCT, as described below. Key steps in the implementation process are summarized in Fig. 2.

#### Exploration (E)

The inclusion of PMTCT in the national HIV strategy was supported by use of local, national, and global data as well as advocacy from healthcare providers. Beginning in 1989, CHUK tested for HIV among pregnant women, providing data on the burden of disease. As research findings on the effectiveness of PMTCT emerged globally, a KI noted that healthcare providers pushed the MOH to include this strategy in the national policy. The goal of PMTCT was to identify HIV-positive pregnant women and children at risk of contracting HIV during the prenatal or delivery period or through breastmilk in postpartum, and to provide interventions to prevent the transmission of HIV from a mother to her child.

#### Preparation (P)

Informed by published international data and protocols and after examining evidence of the burden of HIV among pregnant women at CHUK, the PMTCT program was first piloted in Kigali health facilities in 1999. This program included pretest HIV counseling, routine HIV testing at ANC visits, use of ARVs as prophylaxis for MTCT for HIV-positive pregnant women not eligible for full therapy, and community health education on HIV and PMTCT. To ensure data availability, a health information system to track the number of women in need of and using PMTCT services (TRACnet) was developed. This system employed reporting from mobile phones to allow for timely clinical data utilization [18]. During the 3 years of the pilot phase, 100% of women attending ANC were counseled on HIV testing during their ANC visit, 86% of these women received testing, and 63% of HIV-positive women received ARV prophylaxis [30]. As women traveled across the country to receive these services, the MOH saw the need to scale up this program to reach a wider population.

As a result, in 2001, guidelines for PMTCT were integrated into the existing system. The Treatment and Research on AIDS Center (TRAC) defined national goals for PMTCT in the National Strategic Plan Against HIV, with initial support of the National AIDS Control Commission (NACC) and through the coordination of donor and implementing partners, such as UNICEF and the Elizabeth Glaser Pediatric AIDS Foundation [31]. Additional partners, including PEPFAR and Global Fund, joined the efforts to provide funding for HIV services, including PMTCT, on a larger scale with coordination led by the NACC and the TRAC [32, 33]. To ensure multisectoral coordination, the PMTCT technical working group, comprising members from government agencies, local organizations, and international partners, was formed in 2005. In addition, healthcare providers and local leaders at all levels were engaged through education and sensitization efforts to ensure their understanding and support for PMTCT. Monitoring and evaluation was integrated into the ordinary data management system while ensuring accountability of local leaders through imihigo contracts, a performance management strategy for district mayors [34].

#### Implementation (I)

After the successful pilot testing, Rwanda progressively implemented PMTCT nationally. The national scale-up began in 2006 with the goal of integrating comprehensive PMTCT into Maternal and Child Health (MCH) programming at the level of all health facilities. However, until 2015, ART for PMTCT was only provided at health facilities, while CHWs delivered educative messages to community members [35–37]. The decentralization of PMTCT services to all health facilities contributed to the decline of HIV prevalence among pregnant women, from 9% in 2002 to 1% in 2013 [36]. Quality assurance and adherence to national protocols were ensured through supervision and performance-based financing for healthcare providers, including key HIV care indicators such as the number of HIV-positive pregnant women put on ART during labor [38]. The implementation was steered by national guidelines to which supporting partners nationwide had to align.

#### Adaptation (A)

Data use was key to the adaptation of the PMTCT program. The MOH continually reassessed national policies and guidelines for PMTCT based on emerging national data and international peer-reviewed publications and recommendations. In 2005, the national program changed from single-dose Nevirapine to dual therapy for mothers following the 2004 WHO recommendations for more effective multidrug ARVs [39]. Rwanda’s early adoption of innovation was further evidenced when, in 2010, the MOH adopted Option B to provide ART for all pregnant women for lifelong treatment as well as prevention. This approach would provide the most beneficial treatment regimen to patients, reduce resistance from monotherapy, and prevent future HIV cases [40]. However, the MOH understood that continuing triple therapy indefinitely for all mothers, rather than adjusting treatment after weaning (per WHO’s guidance), would be more beneficial and cost-effective, a strategy later termed Option B+. There was significant pushback from the technical working group, WHO, and donors regarding this approach due to the upfront cost and purported lack of cost-effectiveness. Nonetheless, Rwanda’s leadership remained steady and prepared for this new strategy through collaboration with and guidance from local pediatricians. Rwanda successfully started implementing Option B+ 6 months before WHO adopted it in April 2012 [40]. Later studies have shown that this strategy was in fact more cost-effective in Rwanda for PMTCT, [39] and that it resulted in a national decline in HIV transmission from mothers to infants [41, 42]. This continuous system of learning and improvement allowed Rwanda to consistently remain ahead of the curve and implement the most efficient and effective EBIs.

#### Sustainment (S)

By 2009, all health centers in all districts provided PMTCT services as part of ANC. This integration and investment of PMTCT into ANC has contributed to the sustainment of PMTCT services nationally. In addition, the introduction of task shifting during the same year, with ARV treatment initiated by nurses at the health center level, where the majority of ANC and delivery services were provided, also contributed to the sustainment of PMTCT services and increased the number of providers able to offer full spectrum PMTCT care nationally. In support of this sustainment effort, in 2011, the First Lady of Rwanda launched a campaign to eliminate mother-to-child transmission, thereby making it a national priority [43].

Overall, through the implementation strategies applied across these five steps, Rwanda was able to achieve implementation outcomes such as reach, effectiveness, and fidelity (Table 3).

**Table 3:** Effective prevention of mother-to-child transmission implementation strategies and outcomes

Outcomes	Implementation strategy	Results
Reach	Decentralized scale-up of PMTCT of all health facilities, with clear guidelines and protocols; implementing partner and donor coordination.	In 2015, 92.2% of pregnant women received HIV testing during ANC, and less than 1% were HIV-positive [25, 36]. By 2009, all health centers offered ART, including PMTCT.
Effectiveness and Coverage	Use of monitoring tools and programs to flag and track missed appointments and loss-to-follow-up for individual women and infants; review and update of national guidelines every two years in collaboration with partners and based on evidence.	98% of women coming in for antenatal care tested for HIV; 99% of HIV-positive pregnant women receive ART to reduce MTCT (KI). MTCT rates have dropped from 9.7% in 2006 to 2.4% in 2010 and 1.8% in 2015 [21, 44, 45]. Adoption of improved treatments often happen prior to international recommendations by WHO and other bodies, most notably Option B+.
Feasibility	Integration of PMTCT capacity building into maternal health services and related health systems strengthening, national guidelines, and donor coordination.	Increased ANC services available led to increased availability of PMTCT (KI). The number of health facilities offering PMTCT increased from 53 in 2003 to 382 in 2010 [21].
Fidelity	Monitoring of scale-up and ongoing services, integration into performance-based financing (PBF), clear guidelines supported by training.	See Reach.

*ANC* Antenatal care, *MTCT* mother-to-child transmission, *PMTCT* prevention of mother-to-child transmission

### Contextual factors that facilitated rapid uptake of rotavirus vaccine and PMTCT

We identified a number of contextual factors at the global, national, health system, and community levels that facilitated the rapid and successful implementation of RTV and PMTCT and the adaptation of more effective prevention regimens (Table 4).

**Table 4:** Contextual factors that influenced rotavirus vaccine and prevention of mother-to-child transmission

Level	Contextual factor	Facilitator (+), barrier (−), or both (+/−)	
		RTV	PMTCT
Global	Donor funding priorities and availability	+	+
National/ MOH/ Health system level	Culture of donor and partner coordination	+	+
	Community health system and structure	+	+
	Culture and capacity of data use	+	+
	Financial commitment to the health sector	+	+
	Geographic access to health services	+	–
	In-country research capacity	+	+
	Leadership involvement and a culture of accountability	+	+

#### Donor funding priorities and availability

The preexisting culture of donor and partner coordination at the level of the MOH as well as strong national leadership committed to improving the health sector allowed Rwanda to leverage global priorities (MDGs – reducing U5M), funding for vaccines (GAVI), and HIV financing (Global Fund), and consequently to direct efforts towards addressing major causes of childhood deaths, including diarrhea and HIV [46]. At the global level, the existence of this donor funding allowed Rwanda to rapidly implement the EBIs as soon as evidence of need and effectiveness were shown. This was the case with the RTV, which was approved by the MOH in 2011 and introduced in 2012 with GAVI funding the rollout. Similarly, the existence of donor funding supported the national scale-up of the PMTCT program.

### Culture of accountability, evidence-based decision-making, learning, and improvement

Nationally and at the level of the MOH, Rwanda’s culture of accountability and evidence-based decision-making, learning, and improvement were at the center of health systems strengthening at all levels. The culture of evidence-based decision-making contributed to Rwanda’s rapid uptake of RTV. Rwanda’s decision to adopt this vaccine into the immunization schedule in 2012 followed evidence from 2011 showing the high burden of severe diarrhea caused by rotavirus. The rapid scale-up of PMTCT in 2001, 2 years after the pilot phase in 1999 and following evidence of need and acceptability, demonstrates rapid scale-up based on evidence-based decision-making. Moreover, teams at the district level met monthly to review health center and district-wide data, including RTV coverage, and discuss what was going well and where improvement was needed. This allowed the country both to rapidly adopt EBIs and to adapt as needed.

At the national level, steps to ensure accountability included policies and strategies designed in a participatory process that set out a unified vision and revised decisions based on results and data. At the MOH level, officials performed supervisory visits at every health center, providing feedback on the vaccination campaign, reviewing data regularly, and conducting division-specific meetings to review programs and highlight areas for improvement. This allowed for adequate monitoring of vaccinations (e.g. RTV) and HIV services (e.g. PMTCT). Preparation for PMTCT included engaging with providers and leadership at all levels to ensure understanding and support, integration of monitoring and evaluation through a data system, and building accountability, including through imihigo contracts. Overall, this culture of accountability to the health of the population ensured that the country could adopt effective EBIs rapidly and adapt them (Table 5) as necessary to ensure better health outcomes for the population. This was especially evident from Rwanda’s decision to adapt the Option B+ strategy to include lifelong treatment for mothers, despite opposition from some stakeholders. Based on growing evidence that Option B was more cost-effective than Option A, the existing technical working group initially was not in favor of the change because Option B was more expensive, dependant on donor support, and not yet recommended by the WHO. However, after data review and discussion with the stakeholders, implementers finally agreed that Option B was more beneficial for patients including reducing risk of developing drug resistance, higher effectiveness and offering mothers the opportunity for lifelong therapy. Rwanda ultimately implemented Option B after study findings confirmed its cost-effectiveness for Rwanda, 2 years before the WHO recommended it as Option B+ [40].

**Table 5:** List of evidence-based interventions (EBIs) implemented for reducing U5M in Rwanda

Under-5 cause of death	Evidence-based intervention
Lower respiratory infections	Community-based and facility-based IMCI: pneumonia
	Vaccination: 3 doses of pneumococcal vaccine
	Vaccination: Hib
Diarrheal diseases	Oral rehydration therapy
	Vaccination: 3 doses of rotavirus vaccine
	Community-based and facility-based IMCI: diarrhea
Malaria	Insecticide-treated nets
	Community-based and facility-based IMCI: fever
	Treatment of children with fever by ACT
	Indoor residual spraying
Measles	Vaccination: Measles
HIV	Prevention of Mother-To-Child Transmission (PMTCT)
	ARV treatment for infants and children
	HIV testing of children born to HIV+ mothers
Other vaccine-preventable diseases	Vaccination: 3 doses of DPT, polio, and BCG
Malnutrition	Exclusive breastfeeding for six months
	Continued breastfeeding and complementary feeding after six months
	Vitamin A supplementation
	Management of severe acute malnutrition (ready-to-use food, rehydration, antibiotics)
Neonatal-specific interventions	Antenatal care visits
	Tetanus toxoid vaccination during pregnancy
	Facility-based delivery
	Delivery by skilled birth attendant
	Delivery by Cesarean section when indicated
	Postnatal care

*IMCI* Integrated Management of Childhood Illness, *ACT* Artemisinin-based Combination Therapy

Another challenge was the limited number of medical doctors to deliver PMTCT at all health facilities to ensure full coverage of this care service across all settings. Using task sharing, nurses, who usually provided maternal and child health services at primary healthcare facilities, received a training to provide the PMTCT to pregnant women in their catchment areas. Medical doctors from higher level healthcare facilities and MOH officials conducted mentorship and supervision of the nurses. For rotavirus rollout, challenges identified from both district hospitals and health centers included insufficient refrigerators and cold boxes to distribute vaccines to children whose families live in remote areas. This gap in cold chain capacity was closed through engagement with donors and implementing partners who provided support for the missing equipment.

## Discussion

Between 2000 and 2015, Rwanda made great progress in reducing U5M, from 196 deaths per 1000 live births in 2000 to 50 deaths per 1000 live births in 2015 – a relative reduction of 74% [13]. This success was enabled by the rapid adoption and implementation of EBIs and the use of different strategies adapted to the existing contextual factors. With use of a hybrid implementation research framework, we explored how two EBIs targeted towards the major amenable causes of death among children under 5 were implemented, adapted, and sustained.

Different implementation strategies, including a focus on equity, integration, and donor and implementing partner coordination were used to facilitate delivery of these two EBIs. The success of these strategies was undergirded by the commitment to child health by government leadership, which facilitated effective, rapid implementation and sustainability of the EBIs.

The available funding and the coordination of donors and all development partners greatly facilitated Rwanda’s efforts to strengthen pre-existing systems, such as cold and supply chains for RTV as well as the management and training of health workers. The availability of this funding meant that Rwanda could quickly move from policy adoption to implementation despite domestic resource constraints. This strategy led to the use of funding not only to address disease-specific programs, but also to contribute to horizontal primary care infrastructure development, to reduce the duplication of efforts, and to facilitate rapid scale-up of future vaccines [46].

Investment in the primary healthcare where PMTCT was integrated into ANC has allowed for the sustainment of national PMTCT services that reached expecting mothers in all locations, with the introduction of ARV treatment initiation by nurses at the health center level in 2009 [47]. This task-shifting approach resulted in a significant increase in the number of healthcare providers able to offer full-spectrum PMTCT care nationwide. The strong coordination of health facilities for rotavirus vaccine and PMTCT delivery allowed for successful nationwide reach in all healthcare settings, data collection at all levels (community, health centers, and hospitals), as well as data monitoring, evaluation, and analysis at the MOH level. This strong coordination also informed appropriate strategies for the future introduction of other new vaccines and preventive interventions in Rwanda.

The fact that Rwanda became the first low-income African country to roll out routine RTV reflected the leadership’s understanding of the disease burden, prioritization of child health, adoption of global and national evidence for rotavirus, and ability to learn from previous experiences of vaccine delivery and overall EBI appropriateness. This approach was also used for the PMTCT program. The involvement of Rwanda’s First Lady, Jeannette Kagame, in mobilizing for PMTCT also reflects the importance placed by the government and other leaders in prioritizing and addressing major causes of under-5 deaths, increasing feasibility. In addition, understanding the source and burden caused by pediatric HIV and rotavirus-associated diarrhea in children under 5, the MOH introduced RTV and PMTCT, reflecting a culture of data use that has helped to prioritize the integration of new vaccines into the routine immunization program and the integration of PMTCT into ANC, which also reflect appropriateness. The successful strategy of integrating both PMTCT and rotavirus vaccine into the existing systems reflected feasibility of EBI implementation. Community sensitization and education about the new vaccines and the importance of counseling and HIV testing during ANC, with development of key messaging and dissemination of crucial information throughout the community, were also central to the successful national rotavirus vaccine rollout and establishment of PMTCT services, reflecting acceptability. In addition, equity in the delivery in rotavirus is reflected in both the immunization services which were delivered to all children for free throughout the country and the use of cold boxes and motorcycles to take vaccines to children in remote settings, allowing to achieve a coverage of three doses of more than 90% across subnational regions and all wealth quintiles.

### Limitations

The inability to dive into quality on how the rotavirus vaccine and PMTCT were delivered, and quantification of deaths averted through implementation of these EBIs were limitations for this research. Another limitation is the lack of PMTCT data from the DHS reports, although it was compensated by the TRAC Plus data collected and reported on monthly basis.

#### Key transferable lessons for other settings

This research identified several transferable lessons that could be of value to countries that are still struggling to rapidly adopt and implement EBIs known to be effective elsewhere. These lessons include collection and use of global and national data, engagement with partners for planning and advocacy efforts and for identifying and filling gaps in the cold and supply chains, ensuring accountability and service delivery in all districts, close coordination of partners and stakeholders, integration into the existing health system structure, decentralization, community sensitization and education on new vaccines, and the importance of counseling and HIV testing during ANC.

## Conclusion

Rwanda reduced U5M by 74% between 2000 and 2015, implementing a number of EBIs known to reduce amenable U5M. Despite having scarce resources, the country rapidly adopted and implemented EBIs, including RTV for diarrhea and PMTCT for HIV/AIDS, bridging the gap between research, policy adoption, and practice. This rapid uptake and integration into the health system was achieved through a number of implementation strategies such as donor and implementing partner coordination, integration, decentralization with country-wide delivery, collection and use of data, and regular feedback and improvement mechanisms. The country also leveraged existing facilitators (e.g. national leadership involvement and accountability) and addressed hindering factors (e.g. limited geographic access to health services). Other countries with ongoing challenges with reducing U5M should look into adopting comparable strategies to achieve similar or greater success.

## Data Availability

Quantitate data used in this study can be found on the Exemplars website linked here. Qualitative data access is restricted to users with appropriate ethics approval from the committees listed in the Ethical Considerations section. A reader or reviewer may apply to the authors for access by providing a written description of background, reasons, and intended use. If the methodology does not violate the condition of informed consent under which the interview was conducted, and the proposal approved by UGHE and other relevant ethics boards, the user can obtain the data from the corresponding author, and include one of the authors in the project and analysis.

## Abbreviations

ANC: Antenatal care
ART: Antiretroviral therapy
CHUK: University Teaching Hospital of Kigali
CHW: Community health worker
DHS: Demographic and Health Survey
EBI: Evidence-based intervention
EPI: Expanded Program on Immunization
EPIAS: Exploration, Preparation, Implementation, Adaptation, and Sustainment
FBD: Facility-based delivery
LMIC: Low- and middle-income country
MCH: Maternal and child health
MDGs: Millennium Development Goals
MOH: Ministry of Health
MTCT: Mother-to-child transmission
NACC: National AIDS Control Commission
PCV: Pneumococcal conjugate vaccine
PEPFAR: President’s Emergency Plan for AIDS Relief
PMTCT: Prevention of mother-to-child transmission
RTV: Rotavirus vaccine
TRAC: Treatment and Research on AIDS Center
U5M: Under-5 mortality
